# Enjoying the golden years: social participation and life satisfaction among Chinese older adults

**DOI:** 10.3389/fpubh.2024.1377869

**Published:** 2024-07-31

**Authors:** Min Wu, Dan Yang, Yihao Tian

**Affiliations:** ^1^School of Public Administration, Sichuan University, Chengdu, Sichuan, China; ^2^Social Development and Social Risk Control Research Center of Sichuan Philosophy and Social Sciences Key Research Base, Chengdu, Sichuan, China

**Keywords:** aging, older adults, social participation, life satisfaction, loneliness

## Abstract

**Introduction:**

Older adults commonly face the risk of social isolation, which poses a significant threat to their quality of life. This study explores the association between social participation and life satisfaction among older Chinese adults.

**Methods:**

Data were sourced from the 2018 China Health and Retirement Longitudinal Study. Regression analysis and mediation analysis were employed to examine the relationship between social participation and life satisfaction, with a focus on the roles of loneliness and self-rated health.

**Results:**

The results indicate that social participation is significantly positively associated with older adults' life satisfaction. Furthermore, the positive association is more pronounced with increased diversity in social activities. Mediation analysis reveals that reductions in feelings of loneliness and improvements in health levels mediate the relationship between social participation and life satisfaction. Further analysis showed that social participation had a greater positive association among rural older adults and those lacking family companionship.

**Discussion:**

This study provides evidence for enhancing life satisfaction among older adults and highlights the importance of diversity in social participation.

## 1 Introduction

Population aging is a basic national condition faced by major economies (1). China became an aged society in 2022 and is currently one of the countries experiencing high population aging rates. Data from the National Bureau of Statistics show that, by the end of 2022, the population aged 60 years and above reached approximately 280 million, making up 19.8% of the total population. Such a large older adult population will have a far-reaching impact on various aspects of society and the economy, and corresponding policies must be formulated to cope with the impact of aging (2). It is important to enhance the quality of life of older adults, provide them with better access to services, and enable them to enjoy their golden years peacefully. Life satisfaction is a comprehensive evaluation of an individual's overall life and is a key indicator of mental health and life quality (3), which is a significant reflection of societal progress and development (4). In this context, this study aimed to explore how to improve the life satisfaction of the older adults.

In recent years, an increasing number of scholars have analyzed the influencing factors of the life satisfaction and explored the differences in life satisfaction among different groups (5, 6). Early studies concluded that individuals with high life satisfaction are typically young, well-educated, optimistic, high-income, married, and reside in favorable community environments (6–9). Additionally, studies have examined the impact of social participation on older adults' quality of life and have highlighted the importance of effective social activities in enhancing life quality among the older adults (10). Informal activities such as physical activity and community interactions have been emphasized in studies that investigate enhanced life satisfaction in older adults (10–13), whereas formal activities such as volunteering and club activities are important for older adults' perceptions of self-worth and can enhance their subjective life satisfaction (14, 15). For instance, a Spanish community-based longitudinal survey found that physical activity positively impacted the life satisfaction of older adults (16). Existing literature widely acknowledges that social participation is crucial for older adults to adapt to the changes that occur with aging and to actively embrace the aging process (17, 18). Countries worldwide have implemented various measures, such as the “China 14th Five-Year Plan for Healthy Aging,” which emphasizes promoting healthy aging in China by encouraging older adults to adopt healthy lifestyles and improve their health and quality of life (19). Despite these efforts, the relationship between social participation and life satisfaction among older adults in China remains underexplored. Therefore, we propose hypothesis **H1:** Social participation can positively predict the life satisfaction of older adults.

Social participation also plays a role in reducing loneliness and improving health among older adults. The lack of social interaction can lead to social isolation and increased loneliness, negatively affecting life satisfaction (20–22). Engaging in community activities and maintaining social networks can reduce feelings of loneliness and enhance emotional wellbeing (10, 23, 24). Older adults can reduce feelings of loneliness and enhance their life satisfaction by participating in various forms of social interactions, which can help them understand and realize their values from the spiritual perspective of self-actualization (17). Additionally, social participation is linked to better health outcomes, including reduced depression, improved physical status, and lower mortality rates (25–30). Existing studies also suggest that both physical and psychological health contribute to life satisfaction among older adults (31, 32). Such findings highlight the importance of social participation in maintaining both mental and physical health, as it contributes to overall life satisfaction. We propose the research hypotheses **H2:** Health and loneliness play mediators in the relationship between social participation and life satisfaction among Chinese older adults.

Existing literature acknowledges the differences among various groups of older adults, particularly in terms of urban-rural disparities and variations in family support. There exists conspicuous inequality between urban and rural areas in China, resulting in unfairness between urban and rural residents (33). Urban older adults have more frequent contact with their offspring, easier access to information, and stronger social connections (34–36). Conversely, rural areas suffer from scarce support resources, exacerbating the disparity in health-related living conditions between rural and urban older adults individuals (5, 37–39). It is generally believed that rural older adults individuals experience poorer life quality, while emphasizing the positive impact of social participation on rural older adults individuals (40, 41). Research indicates that rural populations may experience a greater influence from social participation due to their potentially greater need to compensate for emotional deficits through social interaction (42, 43). Social participation also contributes to the life satisfaction of older adults lacking family support (44). Studies indicate that older adults living alone, without family companionship, experience lower life satisfaction and poorer health (45, 46). Widowed individuals may have lower life satisfaction due to the absence of spousal support (47). Intergenerational interactions also influence life satisfaction, with less interaction with younger generations associated with lower satisfaction (48, 49). Therefore, individuals lacking family support rely more on social resources for psychological adjustment and require more social support compared to those with frequent family contact (50). So, we introduce the following research hypotheses H3: **H3a:** Social participation correlates more strongly with life satisfaction for rural older adults compared to urban older adults. **H3b:** Social participation correlates more strongly with life satisfaction for older adults lacking family companionship compared to those with family support.

Building upon the aforementioned research hypotheses, this study utilized data from the 2018 China Health and Retirement Longitudinal Study (CHARLS2018) to investigate the association between social participation and life satisfaction among older adults. Specifically, we focused on two main aspects: first, we explored the relationship between older adults' social participation and their life satisfaction, including potential mediating factors; second, we investigated the variability of social engagement effects across different regions and family contexts among older adults. Through the study of these issues, we hoped to gain insight into the relationship between healthy lifestyles and the quality of life of older adults and explore the characteristics of older adults in terms of emotional and social needs, in order to provide a perspective on experiences and insights which may inform the future design of older adults' services and policies.

## 2 Date and method

### 2.1 Data source

The data used in this study were obtained from the China Health and Retirement Longitudinal Study (CHARLS), which covers approximately 10,000 households and 17,000 individuals in 150 county- and 450 village-level units. CHARLS aimed to collect and analyze high-quality microdata from middle-aged and older adults aged 45 years and older, representing the aging population of China. This study used the cross-sectional data, released in 2018, focusing on individuals aged 60 years and older, to examine the impact of social participation on their life satisfaction. There were 15,941 valid samples in the original data for 2018. The data processing steps in this study are as follows: First, we combined household and individual databases. Second, to retain the sample of our research subjects, we excluded individuals under 60 years old (7,600). Finally, we removed samples with missing primary variables (229), resulting in 8,112 observations being included in the analysis.

### 2.2 Variable measurement

#### 2.2.1 Dependent variable

The dependent variable in this article was the life satisfaction of older adults, which was measured using a questionnaire that asked respondents, “How satisfied are you with your life?” with possible responses of “extremely satisfied, very satisfied, somewhat satisfied, not satisfied, not at all satisfied.” In this study, these five responses were assigned values of 1, 2, 3, 4, and 5, respectively, and the resulting average value was 2.7.

#### 2.2.2 Independent variables

The core independent variable was social participation. A questionnaire was used to ask the respondents if they had engaged in the following 11 social activities in the past month: “visiting friends,” “playing mahjong, chess, or cards, going to the community activity room,” “providing help to relatives, friends or neighbors who do not live with you,” “dancing, exercising, practicing qigong, etc.,” “participating in club activities,” “volunteering or charity work,” “caring for patients or disabled people who do not live with you,” “going to school or attending training courses,” “stock trading (funds and other financial securities),” “using the internet,” and “other social activities.” The 11 options were summarized to measure the older adults' social participation. We examined the richness of social activity participation because it firstly reflects whether individuals actively engage in social activities and secondly represents the extent of social activity involvement. This variable better reflects individuals' social habits. We also considered a dummy variable indicating whether individuals engage in social activities. The purpose of this approach is to allow readers to more intuitively understand the difference in life satisfaction between those who participate in social activities and those who do not, which is a common practice in the literature (51, 52).

#### 2.2.3 Control variables

To control for other factors affecting older adults' life satisfaction, based on an analysis of the literature (6), the study controls for three levels of variables that affect older adults' life satisfaction: individual, family, and social.

At the individual level, we controlled for personal characteristics such as sex, age, education level, religious beliefs, insurance status, employment status, and personal health. At the household level, we controlled for household consumption, residential area, number of household members, whether address has changed, and whether there was a partner. At the social level, we used fixed effects at the community level, such as infrastructure and community services, to control for the effects of inherent community-level characteristics on older adults' satisfaction.

#### 2.2.4 Mediators

The mediation analysis was based on two variables: self-rated health and loneliness. Self-rated health is an ordinal variable of decreasing magnitude, with values from 1 to 5 indicating very healthy, healthy, fair, unhealthy, and very unhealthy individuals, respectively. Self-rated health is widely utilized in the literature and can to some extent reflect individuals' health status (32, 53). Loneliness is an ordinal variable of increasing magnitude, measured by asking how often participants felt lonely in the last week, with values from 1 to 4 being “ < 1,” “1–2,” “3–4,” and “5–7 days.”

### 2.3 Analytical methods and econometric model

We performed statistical analysis using Stata17. Based on the characteristics of the main research variables, this study used ordered probit (Oprobit) regression as the main regression to analyze the data. We conducted mediation analysis using stepwise regression and heterogeneity analysis using grouped regression. We have also employed OLS regression for the main analysis included the results in Supplementary Table 1. To verify the robustness of the analysis, we conducted a propensity score matching (PSM) analysis, with the results reported in Supplementary material A. Oprobit regression is widely used in the literature to deal with cases in which the explanatory variables are ordered, and based on the characteristics of the explanatory variables here, the model was set as follows:


(1) $$Y_{i}^{*}=\alpha +\beta^{*}S_{i}+\gamma^{*}X_{i}+\mu_{c}+\varepsilon_{i}$$

(2) $$Y_{i}=\{\begin{array}{c} 1,Y_{i}{\;}^{*}\leq \mu_{1}\\ 2,\mu_{1}Y_{i}{\;}^{*}\leq \mu_{2}\\ ...\\ ...\\ 5,Y_{i}{\;}^{*}\mu_{4} \end{array}$$

In Equations (1, 2), the ${Y_{i}}^{*}$ is the latent variable of the dependent variable “life satisfaction,” which is unobservable, and *Y*_*i*_ is a non-linear function. The relationship between them is as follows: when ${Y_{i}}^{*}$ is lower than the threshold value μ_1_ the older person is extremely satisfied with life (*Y*_*i*_ = 1); when ${Y_{i}}^{*}$ is higher than μ_1_ but < μ_2_ then the older person is very satisfied with life (*Y*_*i*_ = 2). When ${Y_{i}}^{*}$ is higher than μ_4_ the older person is very dissatisfied with life (*Y*_*i*_ = 5). In Equation (1), *S*_*i*_ is the independent variable “social participation,” *X*_*i*_ includes all control variables at the individual level and household level, and ε_*i*_ is the random disturbance term. Assuming that ε_*i*_ follows a normal distribution across different observations, its mean and variance are standardized to 0 and 1. *Z* denotes all the explanatory variables, and the probability of *Y*_*i*_ can be obtained as shown in Equation (3).


(3) $$\begin{array}{c} P\left(Y_{i}=1\right)=\phi \text{(}\mu_{1}-\beta^{*}Z)\\ P\left(Y_{i}=2\right)=\phi \left(\mu_{2}-\beta^{*}Z\right)\;-\;\phi \left(\mu_{1}-\beta^{*}Z\right)\\ ....\\ P\left(Y_{i}=5\right)=1\;-\;\phi \left(\mu_{4}-\beta^{*}Z\right) \end{array}$$

## 3 Results

### 3.1 Descriptive statistics and regression analysis

**Figure 1:**
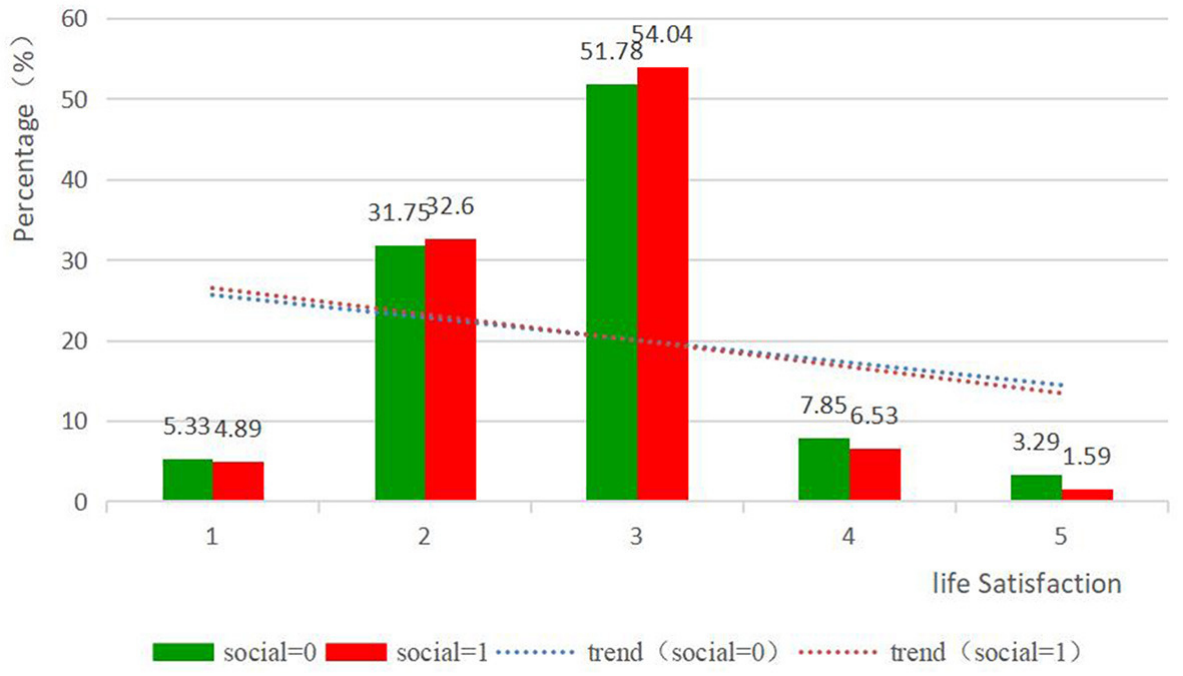
Percentage distribution of life satisfaction.

All variables are presented in Table 1. The sample consists of slightly more males (51.5%) than females (48.5%). Most individuals are aged between 60 and 70 years. A larger proportion of older adults reside in rural areas (58.8%) compared to urban areas (41.2%). Regarding family connections, 18.5% of older adults do not have a partner, and 57.4% are not involved in caring for grandchildren. The number of older adults participating in social activities (50.7%) is comparable to those not participating (49.3%). Figure 1 illustrates that older adults who engage in social activities report lower scores and higher life satisfaction than those who do not.

**Table 1:** Descriptive statistics.

**Variable**	**Introduction**	**Obs**	**%**	**Mean**	**Std. Dev**.	**Min**	**Max**
**Dependent variable**							
Life satisfaction	1–5: extremely satisfied~ not satisfied at all.	8,112		2.700	0.784	1	5
**Independent variable**							
Variety of socialize	Total number of social events	8,112		0.831	1.079	0	9
Socialize	1 = participate socially	4,115	50.7%				
	0 = no social participation	3,997	49.3%				
**Control variables**							
*Individual differences*							
Gender	1 = Male	4,174	51.5%				
	0 = Female	3,938	48.5%				
Age	Continuous Variables	8,112		68.21	6.188	60	108
Education	1–11: Illiterate~ Ph.D.	8,112		3.231	1.914	1	11
Beliefs	1 = Religious	853	10.5%				
	0 = No religion	7,259	89.5%				
Medical insurance	1 = with medical insurance	7,903	97,4%				
	0 = without medical insurance	209	2.6%				
Pension insurance	1 = with pension insurance;	7,119	87.8%				
	0 = without pension insurance.	993	12.2%				
Working status	1 = Be employed	4,402	54.3%				
	0 = Not employed	3,710	45.7%				
Basic diseases	1 = with hypertension	3,738	46.1%				
	0 = without hypertension	4,374	53.9%				
Place of residence	1 = Rural	4,769	58.8%				
	0 = urban	3,343	41.2%				
Change of address	1 = No Changed within 2 years	7,406	91.3%				
	0 = Change within 2 years	706	8.7%				
*Family differences*							
Household consumption	Continuous variables (taking logarithms)	8,112		10.11	0.978	0	14.52
Number of family members	Continuous Variables	8,112		2.650	1.456	1	13
Companionship	1 = with partner	6,612	81.5%				
	0 = without partner.	1,500	18.5%				
Caring for grandchildren	1 = care for grandchildren	3,460	43.6%				
	0 = not care for grandchildren	4,652	57.4%				
**Mediators**							
Self-rated health	1–5: very healthy~ very unhealthy.	8,109		3.031	1.014	1	5
Lonely feeling last week	1: < 1 day; 2: 1–2 days; 3: 3–4 days; 4: 5–7 days.	8,112		1.634	1.048	1	4

Table 2 presents the results of the hierarchical Oprobit regression models that investigated the relationship of social activity participation and life satisfaction in older adults. In column (1–3), they respectively include individual-level control variables, individual and household-level control variables. The regression coefficients continue to show significant negative associations, while the R-squared value gradually increases (β = −0.0339, *P* < 0.01; β = −0.0472, *P* < 0.01; β = −0.0474, *P* < 0.01). This suggests that participating in social activities effectively associated the life satisfaction of older adults, and this positive association becomes more pronounced with greater richness of participation. In column (4–6), the measurement of the dependent variable was changed to whether they engage in social activities, and the correlation still exists (β = −0.0396, *P* < 0.05; β = −0.0590, *P* < 0.05;β = −0.0603, *P* < 0.05), demonstrating the robustness of the results. Furthermore, Table 2 also sheds light on other factors predicting older adults' life satisfaction, Age (β = −0.0186, *P* < 0.01), education (β = −0.0286, *P* < 0.01), work status (β = −0.138, *P* < 0.01), basic disease (β = 0.0785, *P* < 0.01), place of residence (β = 0.743, *P* < 0.01), and companionship (β = −0.114, *P* < 0.01). The results of the VIF analysis for each variable in the analytical model are presented in Supplementary Table 2.

**Table 2:** Regression result of social participation on the life satisfaction of older adults.

		**(1)**	**(2)**	**(3)**	**(4)**	**(5)**	**(6)**
	Variety of socialize	−0.0339^***^	−0.0472^***^	−0.0474^***^			
		(0.0118)	(0.0122)	(0.0122)			
	Socialize				−0.0396	−0.0590^**^	−0.0603^**^
					(0.0262)	(0.0266)	(0.0267)
Individual differences	Age		−0.0155^***^	−0.0186^***^		−0.0151^***^	−0.0182^***^
			(0.00223)	(0.00239)		(0.00223)	(0.00239)
	Gender		−0.0564^**^	−0.0423		−0.0531^*^	−0.0393
			(0.0276)	(0.0279)		(0.0275)	(0.0278)
	Education		0.0269^***^	0.0286^***^		0.0237^***^	0.0256^***^
			(0.00830)	(0.00836)		(0.00822)	(0.00829)
	Belief		−0.0554	−0.0614		−0.0565	−0.0624
			(0.0479)	(0.0479)		(0.0479)	(0.0479)
	Work status		−0.145^***^	−0.138^***^		−0.145^***^	−0.139^***^
			(0.0315)	(0.0316)		(0.0315)	(0.0316)
	Medical insurance		−0.141	−0.132		−0.142	−0.132
			(0.0978)	(0.0980)		(0.0977)	(0.0980)
	Pension insurance		−0.0645	−0.0604		−0.0664	−0.0622
			(0.0431)	(0.0431)		(0.0431)	(0.0431)
	Basic diseases		0.0791^***^	0.0785^***^		0.0804^***^	0.0800^***^
			(0.0258)	(0.0259)		(0.0258)	(0.0259)
	Place of residence		0.754^**^	0.743^**^		0.786^**^	0.771^**^
			(0.331)	(0.328)		(0.331)	(0.328)
	Change address		0.0229	0.0228		0.0243	0.0240
			(0.0474)	(0.0475)		(0.0475)	(0.0476)
Family differences	Companionship			−0.114^***^			−0.113^***^
				(0.0370)			(0.0370)
	Family number			−0.00885			−0.00793
				(0.0105)			(0.0105)
	Caring for grandchildren			−0.0261			−0.0266
				(0.0282)			(0.0282)
	Consumption			−0.0168			−0.0195
				(0.0155)			(0.0154)
	Community Fe	Yes	Yes	Yes	Yes	Yes	Yes
	*N*	8,112	8,112	8,112	8,112	8,112	8,112
	Pseudo *R*^2^	0.045	0.050	0.051	0.045	0.049	0.050

Standard errors in parentheses; ^*^p < 0.1, ^**^p < 0.05, ^***^p < 0.01.

### 3.2 Mediation analysis

**Figure 2:**
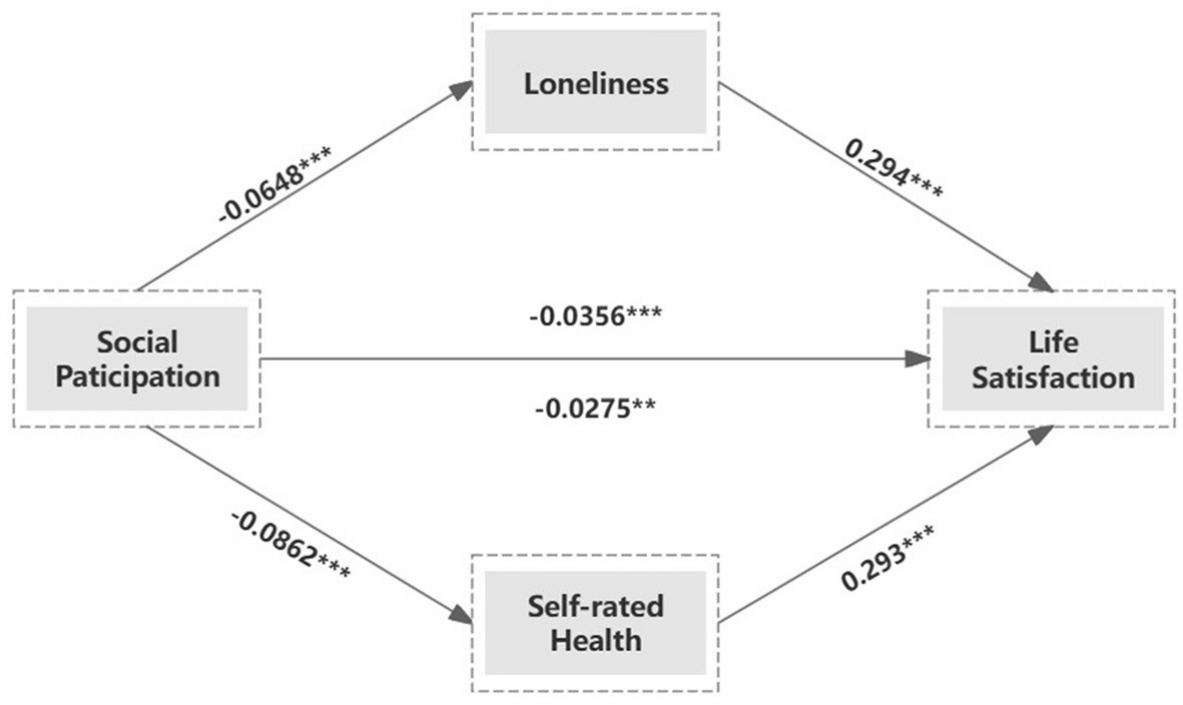
Path diagram of association between social participation and life satisfaction. ^*^p < 0.1, ^**^p < 0.05, ^***^p < 0.01.

The results of the mediation analyses are reported in Table 3. Based on the estimates presented in Table 3 (1–2), social participation predicted lower levels of loneliness among older adults (β = −0.0648, *P* < 0.01) and there was a significant positive relationship between social participation and life satisfaction (β = 0.294, *P* < 0.01). The statistical results shown in Table 3 (3–4) indicate that engaging in social activities enhances self-rated health (β = −0.0862, *P* < 0.01), and self-rated health is significantly positively correlated with life satisfaction (β = 0.293, *P* < 0.01). In both mediation analyses, the absolute value of the correlation coefficients between social participation and life satisfaction (β = −0.0356, *P* < 0.01; β = −0.0275, *P* < 0.05) were smaller than the direct correlation coefficient between social participation and life satisfaction (β = 0.0474, *P* < 0.01). The results of the mediation analysis are visualized in Figure 2.

**Table 3:** Results of mediation analysis.

	**(1)**	**(2)**		**(3)**	**(4)**
**Var**	**Lonely**	**Life satisfaction**	**Var**	**Self-rated health**	**Life satisfaction**
Variety of socialize	−0.0648^***^	−0.0356^***^	Variety of socialize	−0.0862^***^	−0.0275^**^
	(−4.31)	(−2.87)		(−7.25)	(−2.22)
Lonely		0.294^***^	Self-rated health		0.293^***^
		(20.17)			(20.50)
*N*	8,112	8,112	*N*	8,109	8,109
Pseudo *R*^2^	0.089	0.078	Pseudo *R*^2^	0.066	0.077

Standard errors in parentheses; ^*^p < 0.1, ^**^p < 0.05, ^***^p < 0.01.

### 3.3 Heterogeneity analysis

The results, shown in Table 4 (1–2), indicate that there are differences in the positive correlation of social participation with the life satisfaction of rural and urban older adults. The absolute value of correlation coefficient with life satisfaction for rural older adults (β = −0.0603, *P* < 0.01) is greater than that for urban older adults (β = −0.0358, *P* < 0.05). The second examination of heterogeneity explored the association between social participation and life satisfaction among older adults with different levels of emotional support from their families. The findings reported in Table 4 (3–6) suggest a significant correlation between social participation and the life satisfaction of older adults without partners (β = −0.086, *P* < 0.05). In contrast, the relationship between social participation and the life satisfaction of older adults with partners appears to be weaker (β = −0.0356, *P* < 0.05). Among those not providing care for grandchildren, the coefficient of social engagement on life satisfaction was −0.0713, significant at the 1% level. However, for older adults providing care for grandchildren, the coefficient was only −0.034, significant at the 10% level.

**Table 4:** Results of heterogeneity analysis.

**Variable name**	**Residence**		**Family support**			
	**(1)**	**(2)**	**(3)**	**(4)**	**(5)**	**(6)**
	**Rural**	**Urban**	**With partner**	**No partner**	**Caring for grandchildren**	**No caring for grandchildren**
Variety of socialize	−0.0603^***^	−0.0358^**^	−0.0356^**^	−0.0860^**^	−0.0340^*^	−0.0713^***^
	(0.0189)	(0.0175)	(0.0141)	(0.0376)	(0.0196)	(0.0183)
Control variables	√	√	√	√	√	√
Community	√	√	√	√	√	√
Pseudo *R*^2^	0.050	0.055	0.056	0.160	0.093	0.076
*N*	4,769	3,343	6,612	1,500	3,460	4,652

Standard errors in parentheses; ^*^p < 0.1, ^**^p < 0.05, ^***^p < 0.01.

## 4 Discussion

### 4.1 Main findings

Older adults are a vulnerable group in society, and their population will continue to increase for a long time in the future. Improving the life satisfaction of older adults is an inherent requirement and a key point in the development of a global health strategy, and it is of great significance to promote healthy aging.

The study found a significant positive association between participation in social activities and the life satisfaction of older adults. Additionally, the richness of participation in different types of social activities correlates with greater life satisfaction. This finding consistent with Hypothesis **H1** and empirically contributes to the research on the relationship between social activities and life satisfaction (6).

The mediation analysis in our study showed that loneliness and self-rated health partially mediate the relationship between social participation and life satisfaction, with participation in social activities significantly associated with life satisfaction by reducing loneliness and improving self-rated health. We believe that different social activities contribute differently to the older adult population. For example, participating in community activities helps maintain neighborhood relationships among the older adults (9), while volunteering and interest activities broaden their social networks (15, 54), all of which can reduce loneliness and improve mental health. Physical exercise helps older adults improve their physical health (55). The health level of older adults is closely related to their life satisfaction, and good health can bring more life opportunities, a higher quality of life, and higher satisfaction to older adults (56). Both health and loneliness of older adults have been studied as important factors affecting their quality of life (57, 58), which was also verified in the analysis. Our findings support Hypothesis **H2** that health and loneliness mediate the relationship between social participation and life satisfaction. This underscores the importance of encouraging active social engagement among older adults and provides useful references for policies on healthy aging, emphasizing social participation's role in alleviating loneliness and promoting health and life satisfaction.

Our study also provides extensive information on heterogeneity. First, social participation has a greater impact on rural older adults than on urban older adults. This may be due to the relative lack of leisure and entertainment facilities in rural areas compared with cities, and the higher quality of life expectations among older adults in urban areas (59). Simultaneously, it is easier and more convenient for urban older adults to meet their children (60). With the outflow of young and middle-aged people from rural areas in China, the number of empty-nest older adults in rural areas is increasing, making it difficult for them to obtain their sense of life satisfaction from their families (45). Older adults in rural areas are mostly left behind and have fewer face-to-face interactions with their children (37). Therefore, they are more inclined to seek emotional support through external social activities to enhance their life satisfaction. This result supports Hypothesis **H3a**.

Second, participation in social activities has a greater association with older adults who lack family companionship, suggesting that social participation may partially offset the absence of family companionship. On one hand, there is a marginal diminishing effect on the improvement of life satisfaction (61), and older adults with sufficient family companionship may have higher life satisfaction (60). On the other hand, the older adults lacking companionship are more likely to seek emotional support from outside (62), so subjective life satisfaction is more influenced by social activities. This further supports Hypothesis **H3b**, highlighting the need for more attention to be paid to vulnerable older adult populations with emotional deficits, thus aiding them in enjoying their later years.

### 4.2 Policy implications

This study argued that advocating for older adults to participate in social activities and enhance their sense of social inclusion is a key measure for further enhancing their life satisfaction and promoting healthy aging. Therefore, this paper puts forward the following recommendations: first, promote the development of public, cultural, and sports undertakings; improve and enrich relevant services at the community level, such as increasing sports equipment and providing activity venues for older adults; and second, encourage older adults to participate in social activities, such as community volunteer and interest activities, thus reducing the opportunity cost of their social participation. Finally, attention should be paid to older adults in rural areas and those who lack family companionship because their participation in social activities has a significant impact on their quality of life and should be encouraged. In summary, increasing opportunities and choices for older adults to participate in social activities is an important measure for creating an age-friendly society and is of great significance to the development of healthy aging.

### 4.3 Limitations

First, despite the use of multiple methods to enhance the reliability of the results, this was a cross-sectional study owing to data limitations. The panel data allowed us to explore the dynamic changes after participating in social activities. Subsequent research employing panel data will bolster the robustness and reliability of causal inferences. Second, the data for this study were from China; however, the practical situation varies between countries, the utilization of data from diverse countries could enhance the generalizability of this study. Third, the measurement of life satisfaction is self-reported and may be influenced by factors such as social expectations. The adoption of a more objective measurement indicator, if feasible, would be advantageous.

## 5 Conclusion

This study showed that social participation can help older adults reduce their loneliness and improve their self-reported health, thereby improving their life satisfaction. Among all the older adults study participants, those who were lacking family companionship and residing in rural areas showed a stronger association between their life satisfaction and social participation. This study helps us understand the important link between social participation and the older adults' life satisfaction. Future research should continue to discuss the factors that influence the quality of life of older people and make efforts to help them enjoy their golden years.

## Data availability statement

Publicly available datasets were analyzed in this study. The data underlying the results presented in this study are available from the China Health and Retirement Longitudinal Study (CHARLS), and more information on data requests can be found on the CHARLS website: http://charls.pku.edu.cn/.

## Author contributions

MW: Conceptualization, Investigation, Project administration, Resources, Supervision, Validation, Visualization, Writing – review & editing. DY: Conceptualization, Data curation, Formal analysis, Methodology, Software, Writing – original draft. YT: Conceptualization, Formal analysis, Funding acquisition, Investigation, Project administration, Validation, Visualization, Writing – review & editing.
