# Supplementary material for: Enjoying the golden years: social participation and life satisfaction among Chinese older adults
Source: Front Public Health. 2024 Jul 31;12:1377869. doi: 10.3389/fpubh.2024.1377869 (PMC11322126; doi:10.3389/fpubh.2024.1377869)
Supplement: Supplementary file 1 [file Data_Sheet_1.PDF]

Supplementary Material

1 Supplementary A: Results of PSM

In social survey research, we cannot simply compare the life satisfaction of the elderly who participate in social activities with those who do not because there may be inherent differences between the two. Moreover, not all older adults who participate in social activities have higher life satisfaction than those who do not, because the choice of some older adults to participate in social activities may be influenced by personal, family, and other reasons, which leads to self-selection bias in explanatory variables. Based on the characteristics of the sample variables, this study used kernel matching to match each older adult who participated in social activities with the closest older adult who did not. Propensity scores were constructed using probit regression, and the covariates for constructing propensity scores included gender, age, education level, work status, place of residence, whether they have changed addresses, marital status, and whether they take care of grandchildren (1). To ensure that after sample matching, there was no systematic difference between the treatment and control groups in variables other than the main explanatory variable, this study conducted a balance test on the sample, and the sample passed the test. Supplementary Table 3 shows that other explanatory variables affecting older adults' participation in social activities do not have significant systematic differences, and matching effectively reduces self-selection bias in the system.

The correlations between social participation and the life satisfaction of older adults before and after matching under the three matching methods are shown in Supplementary Table 4. The results indicate that after using the PSM matching method and reducing systematic differences due to the sample self-selection problem, there remains a significant positive correlation between social engagement and older adults' life satisfaction, validating the robustness of the regression results.

[1]. Ahmad K, Hafeez M. Factors Affecting Social Participation of Elderly People: a Study in Lahore. *Journal of Animal and Plant Sciences* (2011) 21:

2 Supplementary B: Supplementary Tables

Supplementary Table 1. Regression result of social participation on the life satisfaction of older adults (OLS)

		(1)	(2)	(3)	(4)	(5)	(6)
Individual differences	Social richness	-0.0243***	-0.0324***	-0.0326***			
		(0.00816)	(0.00834)	(0.00837)			
	Socialize				-0.0300	-0.0422**	-0.0432**
					(0.0183)	(0.0185)	(0.0185)
	Age		-0.0105***	-0.0126***		-0.0103***	-0.0124***
			(0.00155)	(0.00166)		(0.00155)	(0.00166)
	Gender		-0.0403**	-0.0305		-0.0381**	-0.0284
			(0.0191)	(0.0194)		(0.0191)	(0.0193)
	Education		0.0155***	0.0166***		0.0134**	0.0146**

			(0.00573)	(0.00577)		(0.00568)	(0.00573)
	Belief		-0.0345	-0.0388		-0.0353	-0.0394
			(0.0335)	(0.0334)		(0.0335)	(0.0334)
	Work status		-0.100***	-0.0959***		-0.101***	-0.0964***
			(0.0220)	(0.0221)		(0.0220)	(0.0221)
	Medical insurance		-0.113	-0.106		-0.114	-0.106
			(0.0709)	(0.0709)		(0.0708)	(0.0709)
	Pension insurance		-0.0422	-0.0395		-0.0435	-0.0407
			(0.0301)	(0.0301)		(0.0301)	(0.0301)
	Hypertension		0.0545***	0.0541***		0.0556***	0.0552***
			(0.0180)	(0.0179)		(0.0180)	(0.0180)
	Rural		0.557**	0.551**		0.579**	0.570**
			(0.240)	(0.237)		(0.240)	(0.238)
	Change address		0.0136	0.0136		0.0147	0.0146
			(0.0330)	(0.0330)		(0.0331)	(0.0331)
Family differences	Companionship			-0.0793***			-0.0788***
				(0.0259)			(0.0259)
	Family number			-0.00624			-0.00565
				(0.00734)			(0.00735)
	Grandchildren care			-0.0197			-0.0200
				(0.0196)			(0.0196)
	Consumption			-0.00988			-0.0117
				(0.0109)			(0.0109)
	Community Fe	Yes	Yes	Yes	Yes	Yes	Yes
	Sample size	8112	8112	8112	8112	8112	8112
	Pseudo R2	0.045	0.054	0.056	0.044	0.053	0.055

Note. Standard errors in parentheses; * $p < 0.1$, ** $p < 0.05$, *** $p < 0.01$

Supplementary Table 2. Variance Inflation Factor (VIF) Test

Variable	VIF	Variable	VIF
Life satisfaction	1.01	Life satisfaction	1.01
Social Richness	1.11	Socialize	1.03
Gender	1.22	Gender	1.21
Age	1.29	Age	1.29
Education level	1.36	Education level	1.33
Beliefs	1.03	Beliefs	1.03
Medical insurance	1.03	Medical insurance	1.03
Pension insurance	1.04	Pension insurance	1.04
Working status	1.28	Working status	1.28
Basic diseases	1.02	Basic diseases	1.02
Place of residence	1.23	Place of residence	1.23

Change of address	1.02	Change of address	1.03
Household consumption	1.22	Household consumption	1.21
Number of family members	1.20	Number of family members	1.20
Companionship	1.17	Companionship	1.17
Caring for grandchildren	1.18	Caring for grandchildren	1.18
Mean VIF	1.15	Mean VIF	1.15

Supplementary Table 3. Sample balance test after kernel matching

	Unmatched	Mean			%reduct	t-test	
Variable	Matched	Treated	Control	%bias	|bias|	t	p>|t|
Age	U	67.86	68.58	-11.70		-5.260	0
	M	67.86	67.91	-0.800	93.10	-0.370	0.711
Gender	U	0.500	0.529	-5.800		-2.590	0.0100
	M	0.500	0.495	1.100	81.60	0.480	0.631
Education	U	3.526	2.926	31.80		14.29	0
	M	3.526	3.466	3.200	89.90	1.410	0.159
Work status	U	0.505	0.581	-15.30		-6.890	0
	M	0.505	0.515	-2.100	86.50	-0.930	0.351
Rural	U	0.532	0.645	-23		-10.36	0
	M	0.532	0.545	-2.600	88.70	-1.160	0.248
Change address	U	0.916	0.909	2.500		1.110	0.265
	M	0.916	0.918	-0.400	84	-0.180	0.854
Companionship	U	0.810	0.820	-2.400		-1.090	0.275
	M	0.810	0.810	0.200	91.40	0.0900	0.926
Care for grandchildren	U	0.454	0.398	11.30		5.070	0
	M	0.454	0.454	-0.100	99.20	-0.0400	0.968

Note. this table shows the balance test of the matched sample after kernel matching. We also performed one to-one nearest neighbor matching and radius matching, both of which reduced the differences in sample characteristics.

Supplementary Table 4. Regression results after matching

	Before matching	Nearest Neighbor Matching	Radius Matching	Kernel Matching
Socialize	-0.0603**	-0.0652**	-0.0600**	-0.0600**
	(0.0267)	(0.0284)	(0.0267)	(0.0267)
Control variables	√	√	√	√
Community	√	√	√	√
Pseudo R2	0.050	0.053	0.050	0.050
Sample size	8112	7302	8105	8105

Note. Standard errors in parentheses; * p < 0.1, ** p < 0.05, *** p < 0.01
